# Supplementary material for: Jacaric Acid Empowers RSL3-Induced Ferroptotic Cell Death in Two- and Three-Dimensional Breast Cancer Cell Models
Source: Int J Mol Sci. 2025 Apr 4;26(7):3375. doi: 10.3390/ijms26073375 (PMC11989411; doi:10.3390/ijms26073375)
Supplement: Supplementary file 1 [file ijms-26-03375-s001.zip › ijms-3485159-supplementary.pdf]

Supplementary Figures

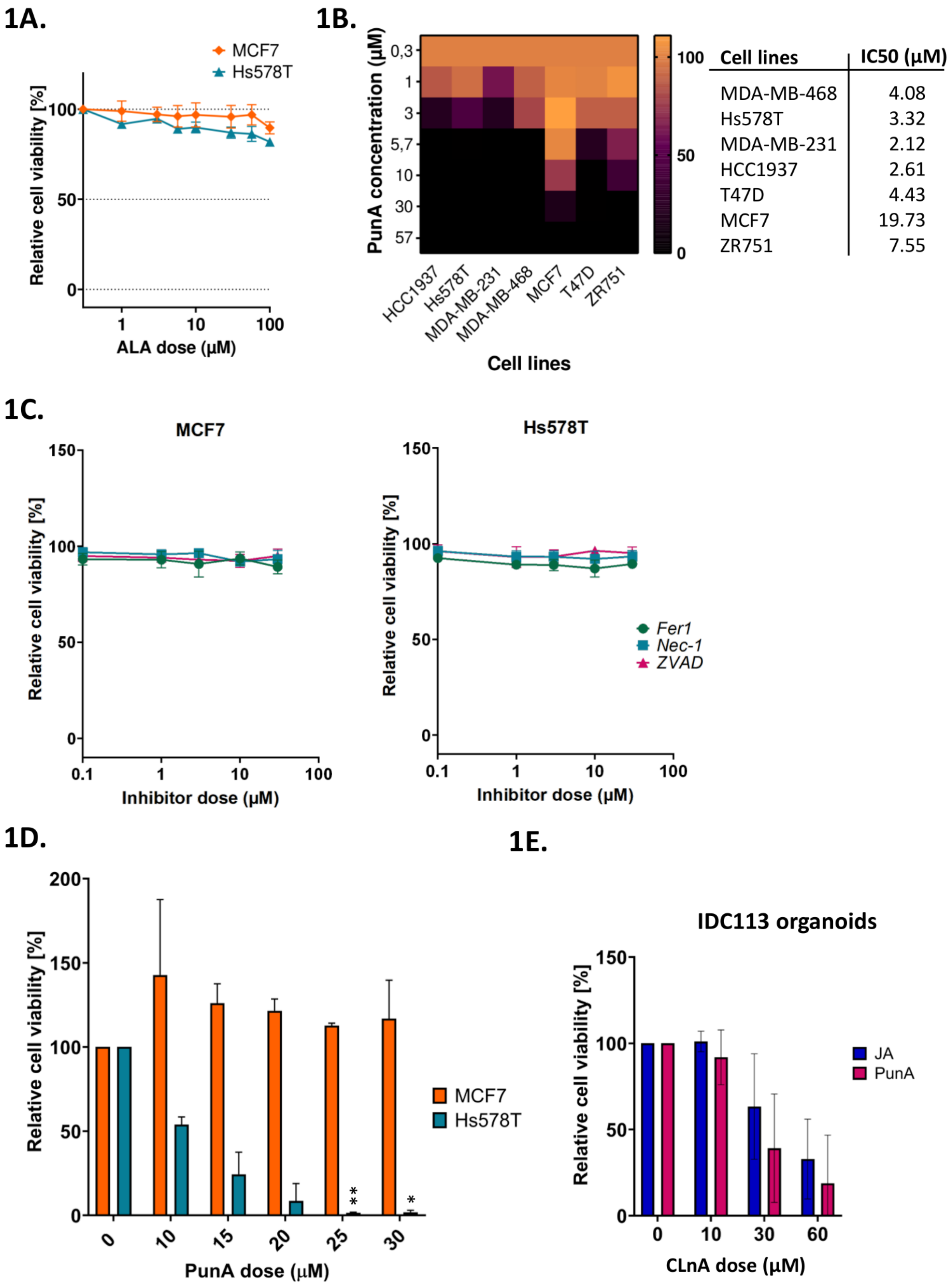

Supplementary Figure S1. Punicic acid (PunA) induces ferroptotic cell death in both two- and three-dimensional-cultured breast cancer models.

(A) Relative viability of Hs578T and MCF7 cells after 72h of treatment with various doses of α-linolenic acid (ALA), normalized to the control (untreated cells). (B) Relative viability of MDA-MB-231, MDA-MB-468, HCC1937, Hs578T, T47D, MCF7 and ZR751 cells after 72h of treatment with different concentrations of PunA, normalized to the control (untreated cells). Associated LD50 values are shown in the table on the right of the graph. (C) Relative viability of Hs578T and MCF7 cells after 72h of treatment with increasing concentrations of ferroptosis, necroptosis and apoptosis inhibitors, namely ferrostatin-1 (Fer1), necrostatin-1 (Nec-1) and Z-VAD-FMK (ZVAD), normalized to the control (untreated cells). (D) Relative viability of Hs578T and MCF7 spheroids treated for 5 days with increasing concentrations of PunA, normalized to the control (untreated cells). (E) Relative viability of IDC113 breast organoids treated with 4 different concentrations of PunA or jacaric acid (JA) for 1 week, normalized

to the control (untreated cells). Results are expressed as mean ± standard deviation of three independent repetitions. Significance was established by two-way ANOVA with Tukey's multiple comparisons (D) or by Kruskal-Wallis test with Dunn's multiple comparisons (C).

Supplementary Figures

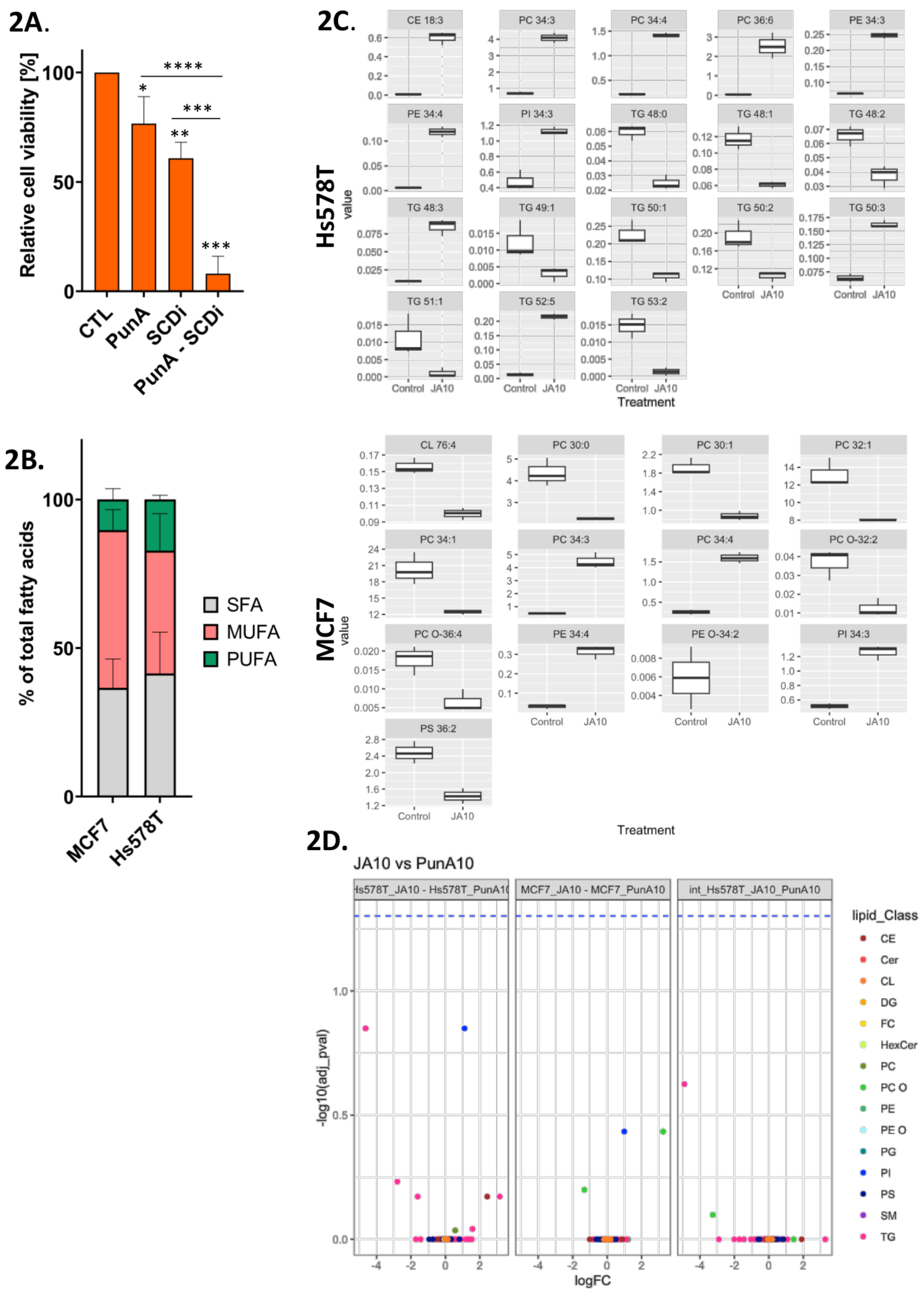

Supplementary Figure S2. Further characterization of lipidomic changes upon conjugated linolenic acid (CLnA) treatment in breast cancer cells.

(A) Relative viability of MCF7 cells treated with either 20 µM of punicic acid (PunA), 10 µM of Stearoyl-CoA desaturase 1 (SCD1) inhibitor or a combination of both treatments, compared to the control (untreated cells). (B) Bar charts showing the repartition of the total fatty acids in MCF7 and Hs578T cells following their degree of saturation: Saturated fatty acids (SFA), Monounsaturated fatty acids (MUFAs), Polyunsaturated fatty acids (PUFAs). (C) Boxplots showing the abundances of the significantly differing lipid species between Hs578T or MCF7 cells treated with 10 µM of JA, and untreated cells. (D) Volcano plots of significance (-log2 of adjusted p-value) and log2 fold change in the abundance of lipid species for Hs578T and MCF7 cells after treatment with 10 µM of JA compared to a treatment with 10 µM of PunA for 4 hours. The 16 lipid

species classes analysed were the followings: FC, PC, PE, PI, PS, PG, TAG, DAG, Cer, hex_Cer, LPC, LPE, PC_O, PE_O, SM and CE. Significance was established by false-discovery rate with Benjamini-Hochberg p-value adjustment for multiple comparisons (D) and by one-way ANOVA with Tukey's multiple comparisons (A). See Figure 2 for other abbreviations.

Supplementary Figures

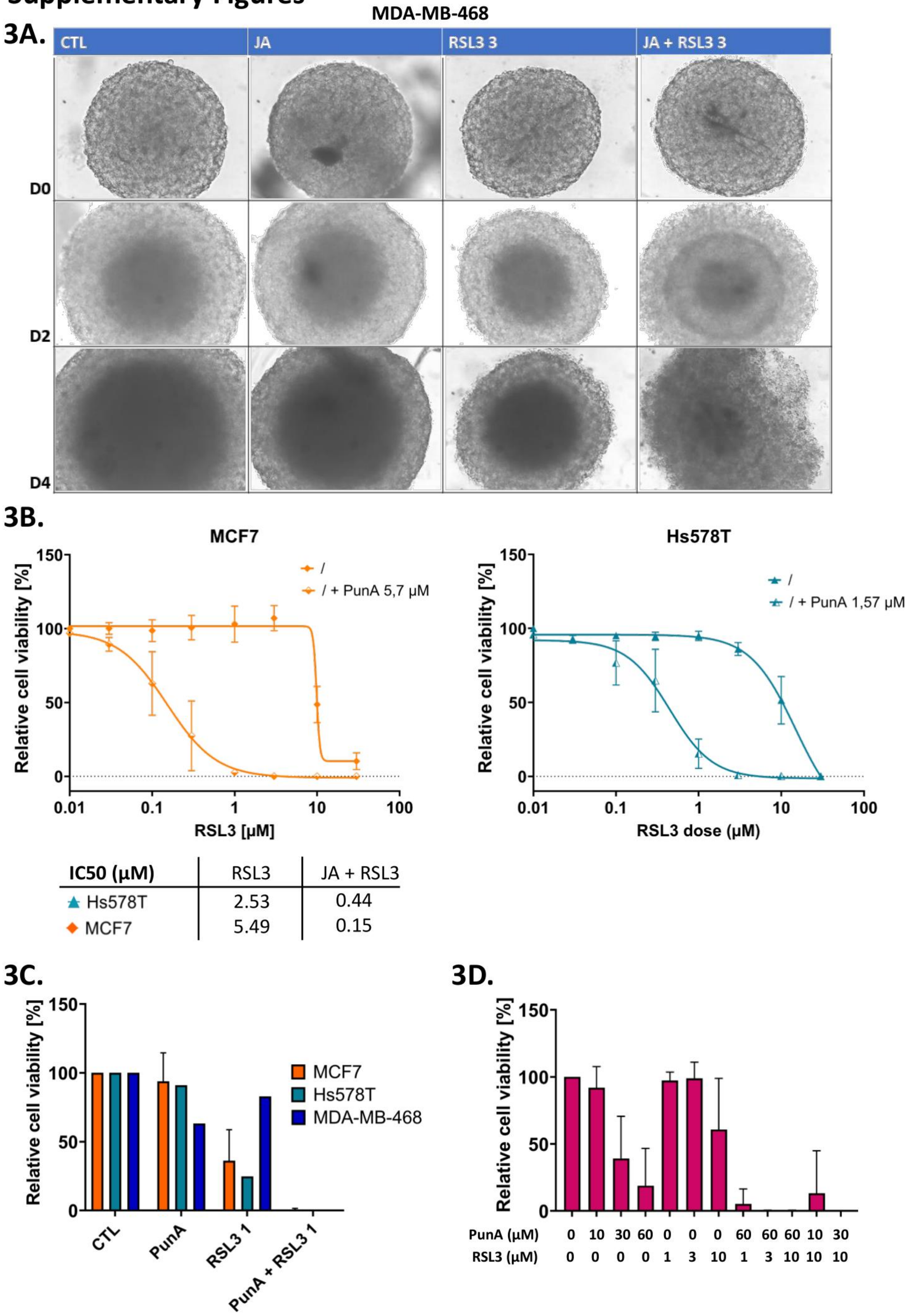

Supplementary Figure S3. Punicic acid (PunA) may empower the efficiency of future cancer treatments as it decreases the dose-efficient concentrations of Ras-selective lethal 3 (RSL3).

(A) Representative pictures of MDA-MB-468 spheroids treated with either 1 µM of jacaric acid (JA) or 1 µM of RSL3 or a combination of both at day 0, 2 and 4. (B) Relative viability of Hs578T and MCF7 cells treated during 72 hours with increasing doses of RSL3, either alone or in combination with a fixed concentration of 1.57 µM for Hs578T or 5.7 µM for MCF7 of PunA, normalized to the control (untreated cells). (C)

Relative viability of spheroids treated for 5 days with 1 µM of PunA for Hs578T and MDA-MB-468 cells and 3 µM for MCF7 cells ± 1 µM of RSL3, normalized to the control (untreated cells). (D) Relative viability of IDC113 breast organoids after 1 week of treatment with increasing concentrations of PunA or RSL3 or a combination of both, normalized to the control (untreated cells). Results are expressed as mean ± standard deviation of the mean of three independent repetitions (B, D).
